# The role of tumour-derived iNOS in tumour progression and angiogenesis

**DOI:** 10.1038/sj.bjc.6606034

**Published:** 2010-12-07

**Authors:** V Kostourou, J E Cartwright, A P Johnstone, J K R Boult, E R Cullis, GStJ Whitley, S P Robinson

**Affiliations:** 1 Division of Basic Medical Sciences, St George's University of London, Cranmer Terrace, London SW17 ORE, UK; 2 Institute of Immunology, BSRC ‘Alexander Fleming’, 34 Fleming Street, Athens 16672, Greece; 3 Cancer Research UK and EPSRC Cancer Imaging Centre, The Institute of Cancer Research and Royal Marsden NHS Trust, Cotswold Road, Sutton, Surrey SM2 5NG, UK

**Keywords:** iNOS, nitric oxide, angiogenesis

## Abstract

**Background:**

Progressive tumour growth is dependent on the development of a functional tumour vasculature and highly regulated by growth factors and cytokines. Nitric oxide (NO) is a free radical, produced both by tumour and host cells, and functions as a signalling molecule downstream of several angiogenic factors. Both pro- and antitumourigenic properties have been attributed to NO.

**Methods:**

The expression of the inducible isoform of NO synthase (iNOS) was knocked down in the C6 glioma cell line using constitutive expression of antisense RNA, and the effect of tumour-derived NO on tumour progression and angiogenesis was investigated.

**Results:**

Tumours in which iNOS expression was decreased displayed significantly reduced growth rates compared with tumours derived from parental C6 cells. Quantitative non-invasive magnetic resonance imaging and fluorescence microscopy of tumour uptake of Hoechst 33342, and haematoxylin and eosin staining, revealed significantly impaired vascular development and function in antisense iNOS tumours compared with control *in vivo*, primarily associated with the more necrotic tumour core. Decreased iNOS expression had no effect on tumour VEGF expression.

**Conclusion:**

Nitric oxide derived from tumour iNOS is an important modulator of tumour progression and angiogenesis in C6 gliomas and further supports the therapeutic strategy of inhibiting iNOS for the treatment of cancer.

Progressive tumour growth and survival is dependent on angiogenesis, the development of a functional blood supply to provide nutrients and remove waste products. Without the concomitant growth of new blood vessels or cooption of existing capillary networks, tumours cannot expand beyond a minimal size or metastasise to distant sites (Folkman, 1995; Carmeliet and Jain, 2000). Angiogenesis is stimulated by the release of specific growth factors from tumour, endothelial, accessory (stromal) host cells and/or associated macrophages, and production of these factors is upregulated by conditions associated with the classical hallmarks of the tumour microenvironment (e.g., elevated interstitial fluid pressure, hypoxia), all of which are consistent with a compromised blood supply (Yancopoulos *et al*, 2000). Although extensively studied, the molecular mechanisms that regulate tumour blood vessel development and function remain elusive, and are a reason for the apparent paucity of potent antiangiogenic therapies.

A plethora of angiogenic stimuli induce the production of nitric oxide (NO), an important inter- and intracellular signalling molecule synthesised from arginine by NO synthases (NOS). Of the three isoforms of NOS, specifically inducible (iNOS), endothelial (eNOS) and neuronal, iNOS produces the greatest amounts of NO and is associated with a number of pathologies (Moncada *et al*, 1991). Although their precise role is still unclear, both iNOS and eNOS activities have been implicated in tumour progression and angiogenesis, and their effectiveness appears to be dependent on their activity and distribution, the concentration and duration of exposure to NO, and the intrinsic sensitivity of cells to NO (Fukumura *et al*, 2006). For example, transfection of K-1735 murine melanoma with iNOS decreased the rate of tumour growth by inducing apoptosis (Xie *et al*, 1995). In contrast, overexpression of iNOS in DLD-1 colon carcinoma cells results in more aggressive and better vascularised tumours compared with wild type (Jenkins *et al*, 1995; Cullis *et al*, 2006). In addition, delivery of iNOS through gene transfer has been shown to both radiosensitise tumours and have direct cytotoxicity *in vivo* (Worthington *et al*, 2004). The results obtained from clinical studies have been more equivocal, revealing positive correlations between iNOS expression and grade in a wide range of human cancers, including brain tumours (Cobbs *et al*, 1995; Ellie *et al*, 1995; Ambs *et al*, 1998). More importantly, increased expression of iNOS in both human breast cancer and melanoma has recently been shown to be associated with poor outcome/survival (Loibl *et al*, 2005; Ekmekcioglu *et al*, 2006).

The effect of altered NO levels on tumour growth and angiogenesis has also been exploited through pharmacological manipulation with either NO donors or NOS inhibitors. Treatment of mice bearing mammary and squamous carcinomas with *N*^G^-nitro-L-arginine methyl ester, a universal NOS inhibitor, as well as specific inhibition of iNOS by either 1400W or L-nil, reduced subcutaneous tumour growth and vessel development (Thomsen *et al*, 1997; Jadeski and Lala, 1999; Sikora *et al*, 2010). Although these observations indicate a tumour-promoting role for NO, the effect of NOS inhibition could not be ascribed specifically to tumour-derived NO, as NOS inhibition can affect other aspects of host physiology. These systemic effects were highlighted during studies in which low concentrations of NO donors inhibited angiogenesis and tumour growth, whereas higher doses of NO donors caused a reduced response because of interference with host physiology (Pipili-Synetos *et al*, 1995). A single dose of the NOS inhibitor *N*^G^-nitro-L-arginine (L-NNA) was shown to induce an acute and sustained reduction in human tumour blood volume, providing clinical evidence that inhibition of NOS has tumour antivascular effects (Ng *et al*, 2007). The importance of host-derived NO for tumour growth has also been shown genetically in studies using iNOS knockout mice, wherein tumour growth and vascularisation were decreased (Konopka *et al*, 2001; Wang *et al*, 2001). However, tumour cells are a major source of NO production that can affect angiogenesis and modulate tumour progression. Therefore, careful examination of the role of tumour-derived NO is important in understanding tumour physiology and in the design of effective therapeutic interventions.

In this study we have investigated the role of tumour cell-derived iNOS on growth and angiogenesis in a model of glioma, which clinically represents one of the most vascularised human cancers (Wesseling *et al*, 1997). The expression of iNOS in C6 glioma cells was reduced using an endogenous antisense approach, and the growth potential of these antisense-iNOS cells was examined *in vivo*. Furthermore, the functional blood supply of tumours derived from these manipulated cell lines was investigated *in vivo* using non-invasive magnetic resonance imaging (MRI), and complemented with fluorescence microscopy.

## Materials and methods

### Cell culture and transfection

The rat glioma C6 cell line (European Collection of Cell Cultures, Salisbury, UK) was maintained in Nutrient Ham's F-10 (Sigma, Dorset, UK) culture medium containing 2 mM L-glutamine, 100 U ml^−1^ penicillin, 0.1 mg ml^−1^ streptomycin and 10% (v/v) fetal calf serum. The antisense iNOS stable-transfected cell lines (subsequently termed AS lines) were produced by transfection with the pciNOS500 plasmid using the poly L-ornithine method (Kostourou *et al*, 2002) and selected by culture in hygromycin (500 *μ*g ml^−1^). For the generation of the pciNOS500 plasmid, the fragment of murine iNOS (5707–6247 bp), previously shown to be an effective antisense target (Cartwright *et al*, 2000), was extracted from the original clone in pSG5 vector using *BamHI* restriction enzyme and subcloned into the *BamHI* site in pcDNA 3.1 (+)/hygro vector (Invitrogen, Paisley, UK).

### Western blot analysis

Cell extracts were generated from stably transfected antisense cell lines (AS7, AS9 and AS12) and parental C6 cells with cytokine stimulation (10 ng ml^−1^ TNF-*α*, 100 U ml^−1^ IFN-*γ*, 5 *μ*g ml^−1^ LPS for 24 h), and from tumour homogenates of AS7 and C6 tumours (Kostourou *et al*, 2002). Protein concentration was measured by the Bradford method according to the manufacturer's instructions (Sigma, Poole, Dorset, UK). An equal amount of protein was analysed by SDS–PAGE and the iNOS protein was detected using a polyclonal anti-mouse iNOS antibody (M-19, Santa Cruz, Heidelberg, Germany). Equal protein loading was verified on tumour homogenate blots using a monoclonal anti-*β*-actin antibody (AC-15, Abcam, Cambridge, UK).

### Nitrite production

C6, AS7 and AS12 cells were stimulated with 10 ng ml^−1^ of TNF-*α*, 100 U ml^−1^ of IFN-*γ* and 5 *μ*g ml^−1^ of LPS for up to 72 h and the nitrite production was determined using the Greiss reaction as previously described (Kostourou *et al*, 2002).

### *In vitro* growth

The growth of C6 and AS7 cells under normal culture conditions or after cytokine stimulation (5 *μ*g ml^−1^ LPS, 100 U ml^−1^ IFN-*γ* and 10 ng ml^−1^ TNF-*α*) was determined using a dye-binding assay, in which the concentration of the eluted dye is linearly correlated to the cell number (Winterbourne, 1993). AS7 and C6 cells were seeded at a density of 1000 cells per well in 24-well plates. Fresh medium alone or with addition of cytokines was added every 2 days. Cell number was determined in duplicate wells at daily intervals for a period of 6 days. The cells were fixed in 300 *μ*l fixation solution (10% (v/v) formalin in 5% (w/v) polyvinylpyrrolidone) for 5 min. The wells were then washed three times with water and air-dried. The cells were stained with 600 *μ*l of filtered 0.04% (w/v) Coomassie Blue R250 (Sigma) in 25% (v/v) ethanol/12% (v/v) acetic acid for 1 h, washed three times with 600 *μ*l of 10% (v/v) ethanol/5% (v/v) acetic acid and allowed to dry. Cell growth was quantified by eluting the stain in 600 *μ*l filtered solution of 1 M potassium acetate in 70% ethanol for 1 h and measuring absorbance at 620 nm using a Titertek Multiscan microtitre plate reader (Titertek, McLean, VA, USA).

### Animals and tumours

All procedures were performed in accordance with the local ethical review panel, the UK Home Office Scientific Procedures Act 1986 and the UKCCCR guidelines (Workman *et al*, 2010). Female (7–8 weeks old) MF1/nu mice were injected subcutaneously in the flanks with AS7, AS12 or C6 cells in 0.1 ml PBS. Tumour growth was measured at daily intervals after briefly anaesthetising the mice with fluothane in 2% N_2_O, 5% O_2_. The dimensions of the tumour under the skin were measured using calipers and tumour volume was calculated using the following formula: tumour volume=*π*/6· (length × width × depth).

### Magnetic resonance imaging

Fractional tumour blood volume was evaluated *in vivo* using susceptibility contrast MRI (Robinson *et al*, 2003). Intravascular contrast agents such as ultrasmall superparamagnetic iron oxide (USPIO) particles create magnetic susceptibility variations inducing an increase in the MRI transverse relaxation rate *R*_2_^*^ (s^−1^) of water in the surrounding tissue. Changes in tissue *R*_2_^*^ caused by USPIO particles are dependent on blood volume; hence, an estimate can be made by measuring the absolute changes in *R*_2_^*^ following administration of USPIO particles. As USPIO particles probe for blood vessels that are functional at the time of injection, and not necrotic areas or vessels with static blood flow, a USPIO-induced measurement of Δ*R*_2_^*^ can provide a valuable non-invasive imaging biomarker associated with tumour angiogenesis (Kostourou *et al*, 2003; Robinson *et al*, 2008). A multigradient echo (MGRE) MRI sequence was used to quantify *R*_2_^*^ of size-matched (∼0.8 cm^3^) AS7 and C6 tumours before and after the intravenous administration of 2.5 mg Fe kg^−1^ of the USPIO contrast agent feruglose (GE Healthcare, Oslo, Norway) through a lateral tail vein. Apparent *R*_2_^*^ maps were calculated on a voxel-by-voxel basis from the MGRE image data sets, and the average apparent *R*_2_^*^ relaxation rates were calculated for each tumour for a region of interest encompassing the whole tumour image but excluding any surrounding skin and muscle. Having determined the Δ*R*_2_^*^ values (*R*_2_^*^_post-USPIO_−*R*_2_^*^_pre-USPIO_), estimates of the fractional tumour blood volume (*ξ*, %) were then derived as previously described (Robinson *et al*, 2003, 2008).

### Immunohistochemistry and fluorescence microscopy

Mice bearing size-matched AS7 and C6 tumours were injected intravenously with 15 mg kg^−1^ of the perfusion marker Hoechst 33342 (Sigma) 1 min before excision of the tumour (Kostourou *et al*, 2003; Cullis *et al*, 2006). Three acetone-fixed cryosections (8–10 *μ*m) of each C6 and AS7 tumours were then visualised for Hoechst 33342 uptake by fluorescence microscopy using appropriate filters (355 nm excitation, 465 nm emission). To assess vessel density, additional frozen tumour sections were first rehydrated with PBS, and then incubated with 2% BSA/5% goat serum in PBS for 30 min at room temperature. After washing with PBS, sections were incubated with rat anti-mouse PECAM monoclonal antibody (1 : 500 dilution, BD Pharmingen, Basingstoke, UK) for 1 h at room temperature, and then washed extensively in PBS/0.1% Tween-20. Biotinylated anti-rat immunoglobulins (1 : 100, Vector Laboratories, Burlingame, CA, USA) were then applied onto the sections and incubated for 1 h at room temperature. Following extensive washing in PBS/0.1% Tween-20, sections were incubated with fluorescein streptavidin (1 : 100) in the dark for 30 min, washed with PBS/0.1% Tween-20 and mounted using Vectashield mounting medium (Vector Laboratories). Endothelial structures were subsequently visualised by fluorescence microscopy at 450–490 nm. Whole tumour sections were scanned using a computer-controlled motorised scanning stage (Marzhauser, Wetzlar-Steindorf, Germany) driven by Stage-Pro (Media Cybernetics, Wokingham, UK) on an IX70 fluorescence microscope (Olympus Optical, London, UK). Digital images from both AS7 and C6 tumour sections were acquired using the same exposure time and composite images were synthesised. The digital images were analysed using analySIS software (Soft Imaging System, Munster, Germany). Fluorescent particles were detected above a constant threshold for all the composite images, and the area of the tumour section with fluorescein and Hoechst 33342 fluorescence was determined and expressed as a percentage of the whole tumour section. Additional formalin-fixed tumour sections were cut and stained with haematoxylin and eosin to assess tissue viability of AS7, AS12 and C6 tumours.

### Production and detection of VEGF

Culture medium was collected from confluent six-well plates containing C6 and AS7 cells under normal culture conditions or after cytokine stimulation, and analysed for VEGF_165_ expression using a murine VEGF ELISA kit (R&D Systems, Abingdon, UK). In addition, tumours were excised, weighed, cut into small pieces and maintained in culture medium for 4 days, following which the secretion of VEGF_165_ by the tumour explants into the culture medium was also determined by ELISA (Kostourou *et al*, 2002).

## Results

### Inhibition of iNOS expression in C6 glioma cells

**Figure 1:**
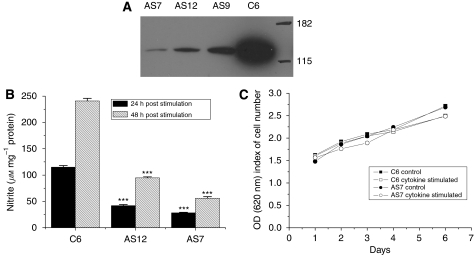
Characterisation of antisense iNOS cell lines *in vitro*. (**A**) Cytokine-induced (10 ng ml^−1^ TNF-*α*, 100 U ml^−1^ IFN-*γ*, 5 *μ*g ml^−1^ LPS for 24 h) iNOS expression from parental C6 and antisense iNOS clones AS7, AS9 and AS12 determined by western blot. (**B**) Nitrite production in parental C6 and antisense iNOS clones AS7 and AS12 determined 24 and 48 h after cytokine stimulation. Results are mean±1 s.e.m. of triplicate of three separate experiments, ^***^P<0.001, Student's *t*-test. (**C**) *In vitro* growth rate of parental C6 and AS7 cells under normal culture conditions or after cytokine stimulation (10 ng ml^−1^ TNF-*α*, 100 U ml^−1^ IFN-*γ* and 5 *μ*g ml^−1^ LPS). Results are mean±1 s.e.m. of triplicate of three separate experiments. Note that decreased iNOS expression does not affect *in vitro* tumour cell growth and survival.

A number of hygromycin-resistant C6 cell lines were isolated following transfection with plasmid pcDNA 3.1 expressing a 500 base fragment of murine iNOS cDNA in an antisense orientation. The efficacy of this fragment to inhibit the expression of iNOS in these lines was determined by western blot analysis. Under basal conditions, the expression of iNOS by C6 cells was undetectable. On stimulation with TNF*α*, IFN*γ* and LPS for 24 h, iNOS expression was increased (Figure 1A). In cells expressing antisense iNOS, there was variable but significant inhibition of iNOS expression. The most significant reduction in iNOS expression was exhibited by clones AS7 and AS12, and these lines were chosen for further investigations. The decrease in iNOS protein expression was corroborated by the reduced NO production by AS7 and AS12 cells, as determined by measuring the accumulation of nitrite following stimulation with TNF*α*, IFN*γ* and LPS for 24 h. The AS7 and AS12 clones displayed significant inhibition of NO production 24 h after cytokine stimulation (76 and 63%, respectively), compared with parental C6 cells, and this level of inhibition of iNOS activity remained similar at the later time point of 48 h (Figure 1B). The reduction in iNOS expression did not alter the *in vitro* growth properties of C6 cells. The basal- or cytokine-stimulated survival of AS7 cells was no different from that of parental C6 cells (Figure 1C).

### Effect of inhibiting iNOS expression on tumour growth *in vivo*

**Figure 2:**
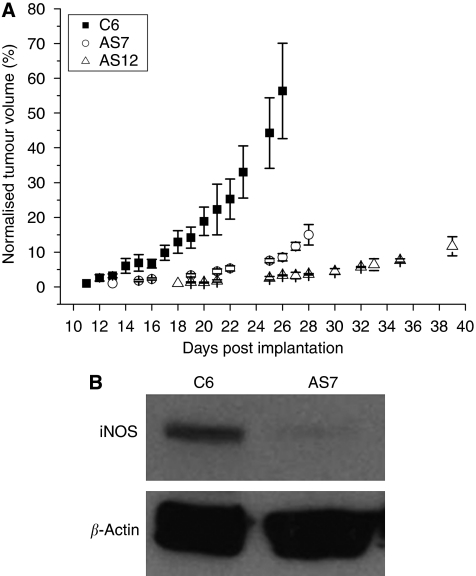
Effect of antisense iNOS on tumour growth *in vivo*. (**A**) Normalised growth rates of tumours derived from parental C6 (*n*=6), AS7 (2 × 10^6^, *n*=5) or AS12 (1 × 10^6^, *n*=5) cells, grown subcutaneously in the flanks of female nude mice. Data points are mean±1 s.e.m. Note the significantly slower growth rate of AS7 and AS12 tumours. (**B**) Western blot of AS7 and C6 tumour homogenates, showing reduced expression of iNOS in the AS7 tumours.

In contrast to their growth *in vitro*, when AS7 or AS12 cells were inoculated into the flanks of nude mice, the tumour growth rate *in vivo* was significantly slower (AS7 doubling time of ∼5 days) than that of C6 tumours (doubling time of ∼4 days, Figure 2A). Tumours derived from AS12 cells exhibited a growth rate similar to that of AS7 tumours. AS7 tumours became palpable and measurable 13 days post inoculation of cells compared with C6 tumours, which could be measured 10 days post inoculation. After 20 days of growth, the mean tumour size of AS7 tumours was half that of C6 tumours. Inhibition of iNOS expression in AS7 tumours was confirmed by western blot analysis of tumour homogenates (Figure 2B).

### Effect of inhibiting iNOS expression on tumour vascular development

**Figure 3:**
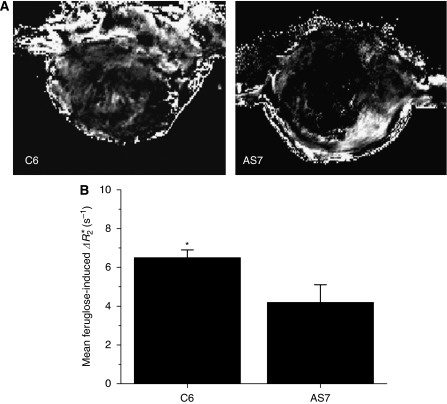
Effect of antisense iNOS on tumour blood volume assessed by MRI. (**A**) Calculated Δ*R*_2_^*^ maps of C6 and AS7 tumours. Note the dark core within the core of the map of the AS7 tumour, indicating an absence of feruglose uptake within these regions. (**B**) Summary of feruglose-induced Δ*R*_2_^*^ of parental C6 (*n*=6) and AS7 (*n*=7) tumours. Results are mean±1 s.e.m. ^*^P<0.05, Student's *t*-test.

Susceptibility contrast-enhanced MRI using the USPIO contrast agent feruglose was used to interrogate any differences in magnitude and distribution of fractional blood volume between size-matched C6 and AS7 tumours. Calculated Δ*R*_2_^*^ maps of C6 and AS7 tumours are shown in Figure 3A. Visual inspection of the tumour Δ*R*_2_^*^ maps consistently revealed that feruglose uptake was mainly restricted to the tumour periphery of AS7 tumours, compared with the more heterogeneous Δ*R*_2_^*^ maps of C6 tumours. Administration of feruglose significantly increased *R*_2_^*^ of the C6 tumours only (Figure 3B). The mean fractional tumour blood volume (*ξ*) derived from these data was 1.4±0.1% and 0.9±0.2% for C6 and AS7 tumours, respectively.

**Figure 4:**
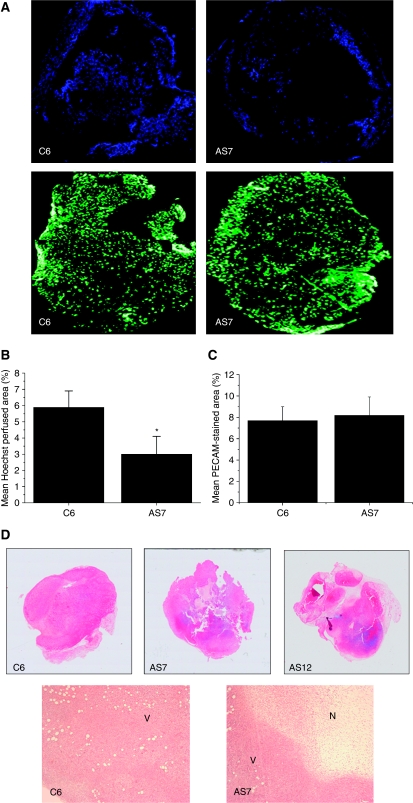
Assessment of tumour perfusion by fluorescence microscopy. (**A**) Composite fluorescence images of Hoechst 33342 uptake, and PECAM staining, in whole C6 and AS7 tumour sections. Note that Hoechst 33342 uptake is restricted to the periphery of the AS7 tumour. (**B**) Quantitation of Hoechst 33342 uptake (mean Hoechst perfused area, %) obtained from three sections each of C6 (*n*=5) and AS7 (*n*=4) tumours. (**C**) Quantitation of vascular density (mean PECAM stained area, %) obtained from sections of C6 (*n*=6) and AS7 (*n*=4) tumours. Results are mean±1 s.e.m., ^*^P<0.05, Student's *t*-test. (**D**) Composite images of whole haematoxylin and eosin-stained sections of tumours derived from C6, AS7 or AS12 cells, and localised images ( × 40 magnification) acquired from within the same sections. Parental C6 tumours exhibited homogeneous, viable (V) tissue, whereas the AS7 and AS12 tumours presented with increased central necrosis (N) and a reduction of viable tissue.

Tumour perfusion was also assessed by quantifying the uptake of the fluorescent perfusion marker Hoechst 33342. This is a nuclear dye that, on injection, stains endothelial cells and cells immediately adjacent to tumour blood vessels. The time from injection to euthanisation was limited to 1 min to prevent excessive diffusion of the dye beyond the cells lining perfused blood vessels within the tumour. Thus, Hoechst 33342 uptake reports only on functionally perfused vessels, and also provides validation of the MRI data. Composite images of Hoechst 33342 uptake showed that, as with MRI data, AS7 tumours had fewer perfused areas compared with C6 tumours, and that these regions were restricted mainly to the tumour periphery (Figure 4A). The perfused vessels were quantified using image analysis techniques and the mean perfused area of AS7 tumours was significantly lower compared with that of C6 tumours (Figure 4B). In comparison, the pan-endothelial marker PECAM was more homogeneously and extensively distributed across both C6 and AS7 tumours, with no significant difference in vessel density between the two tumour types (Figure 4A and C). Composite images of whole haematoxylin and eosin-stained sections of C6, AS7 and AS12 tumours are shown in Figure 4C. Essentially, the darker stain is indicative of more viable tissue. Histological examination of the sections showed that AS7 and AS12 tumours exhibited a high degree of tissue heterogeneity, central necrosis and reduction of viable tissue, consistent with insufficient perfusion, compared with the more homogeneous and viable parental C6 gliomas.

### Decreased AS7 tumour perfusion is not mediated by decreased VEGF expression

**Figure 5:**
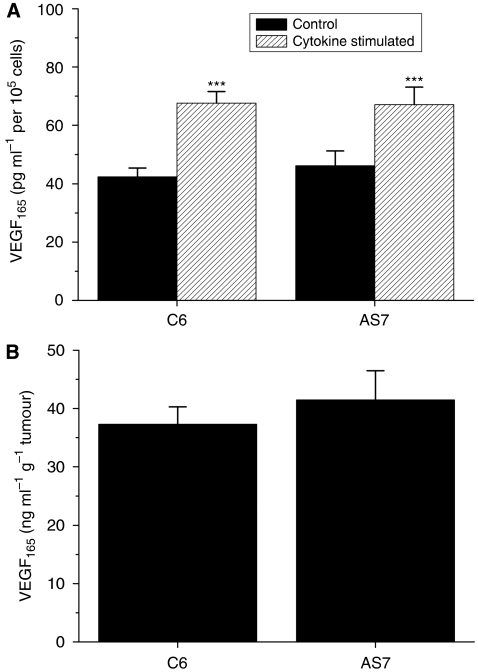
Effect of antisense iNOS on VEGF expression *in vitro* and *ex vivo*. (**A**) VEGF_165_ production by parental C6 and AS7 cells under normal culture conditions or 24 h after cytokine stimulation (10 ng ml^−1^ TNF-*α*, 100 U ml^−1^ IFN-*γ* and 5 *μ*g ml^−1^ LPS). Results are mean±1 s.e.m. of duplicate of three separate experiments. ^***^P<0.001, Student's *t*-test. (**B**) The VEGF_165_ production by C6 and AS7 tumour explants. Results are mean±1 s.e.m. of triplicate of two separate experiments.

Nitric oxide has also been shown to both stimulate and inhibit VEGF expression in a cell-dependent manner and is a potent modulator of vascular development and tumour progression (Chin *et al*, 1997; Tsurumi *et al*, 1997; Liu *et al*, 1998; Yancopoulos *et al*, 2000). *In vitro* studies of AS7 and C6 cells showed that both cell lines produced similar levels of VEGF_165_. Induction of iNOS with cytokines for 24 h resulted in a significant 1.5-fold upregulation of VEGF_165_ in both AS7 and C6 cell lines (Figure 5A). In addition, the concentration of VEGF_165_ in the medium of tumour explants exhibited no significant differences between AS7 and C6 tumours, as determined by ELISA (Figure 5B).

## Discussion

The effect of NO, and in particular tumour cell-derived NO, on tumour growth and angiogenesis is still unclear. Numerous preclinical studies have implicated NO in both tumour growth and angiogenesis *in vivo* with controversial conclusions (Jenkins *et al*, 1995; Thomsen *et al*, 1997; Ambs *et al*, 1998; Jadeski and Lala, 1999; Camp *et al*, 2006; Cullis *et al*, 2006; Sikora *et al*, 2010). The majority of these studies used pharmacological manipulation of NO with either NO donors or inhibitors. There are, however, several disadvantages with this approach, including the lack of NOS isoform selectivity of existing inhibitors, and their systemic effects on host physiology. In addition, NO donors can rapidly generate high levels of NO, together with other cytotoxic molecules such as cyanide, reactive nitrogen intermediates, peroxynitrite and superoxide, making it impossible to dissect the role of NO from the cytotoxicity caused by other reactive species (Borutaite *et al*, 2000). *In vivo,* cells are more likely to experience lower concentrations of NO over prolonged time periods.

In this study, an alternative approach was taken that aimed to overcome some of these shortcomings. Instead of overexpressing the iNOS isoform, which could result in non-physiological, extremely high levels of NO, the role of iNOS on tumour growth and angiogenesis was studied by more subtly decreasing endogenous iNOS expression using antisense technology. Rat C6 glioma cells, which express iNOS, were used, as tumours derived from them represent a well-established model of human glioblastoma (Simmons and Murphy, 1992; Barth, 1998). Furthermore, positive correlations of malignancy with iNOS expression have been shown in human brain tumours (Cobbs *et al*, 1995; Ellie *et al*, 1995), which typically present as highly vascularised tumours in the clinic (Wesseling *et al*, 1997). Although the growth rate of these cells *in vitro* was unaltered, tumours derived from the iNOS-antisense-transfected C6 cell lines displayed significantly reduced growth *in vivo* compared with tumours derived from wild-type C6 cells. Compared with control, cytokine-stimulated AS7 and AS12 clones exhibited a clear reduction in iNOS expression and nitrite production *in vitro*, and reduced iNOS expression was found in AS7 tumours.

Together, these data emphasise the importance of tumour cell-derived iNOS in the progression of C6 gliomas. The absence of a substantial difference in iNOS expression *in vivo* presumably reflects contributions of iNOS from accessory cells, especially macrophages, within solid tumours. Propagation and interrogation of tumours from these clones in iNOS knockout mice should provide an even more unambiguous assessment of the contribution of tumour cell-derived iNOS. Using a similar antisense approach, Yamaguchi *et al* (2002) reported that decreased iNOS expression resulted in reduced glioma formation in an orthotopic rat C6 striatal implantation model. However, the mechanism for the failure of tumour growth was not clarified. It was speculated that decreased expression of iNOS in tumour cells may render them more sensitive to the host immune response. Here we provide evidence for an alternative mechanism by which tumour-derived NO affects tumour growth. Determination of fractional tumour blood volume by non-invasive susceptibility contrast MRI, and histologically qualified with fluorescence microscopy of Hoechst 334342 uptake, revealed that decreased tumour growth is a result of a dysfunctional vascular network that did not support effective tumour perfusion, particularly within the tumour core.

To accommodate sustained tumour growth, the vasculature has to undergo constant remodelling. As a part of this process and as a result of the elevated interstitial fluid pressure (IFP) associated with tumours, and because of local imbalances of angiogenic factors and or intermittent blood flow, some vessels within the tumour may collapse and cease to function. Although these vessels will stain positive for endothelium and contribute to the vessel density measurements, they will not necessarily contribute to tumour growth (Eberhard *et al*, 2000). We therefore used susceptibility contrast MRI *in vivo* and fluorescence microscopy of Hoechst 33342 uptake to assess the degree and distribution of any functional differences in the perfusion of vessels derived from the two cell types *in vivo*, and showed that inhibition of iNOS resulted in reduced tumour perfusion primarily restricted to the tumour periphery. Nitric oxide is a potent mediator of tumour vascular tone (Fukumura *et al*, 2006). Sustained functional tumour perfusion, assessed by Hoechst 33342 uptake, within the core of DLD-1 colon carcinomas derived from cells constitutively overexpressing iNOS, has been reported, and it was hypothesised that the increased concentration of NO may abrogate any IFP-induced vascular collapse (Cullis *et al*, 2006). Similarly, inhibition of iNOS may facilitate IFP-induced vascular collapse, resulting in the absence of functional tumour perfusion within the AS7 tumours reported herein.

Several factors have been implicated in tumour development, one of the most prominent being VEGF, which induces sprouting angiogenesis, increases blood vessel permeability and maintains tumour vessel integrity (Yancopoulos *et al*, 2000). The expression of VEGF has been shown to be a survival factor for blood vessels in C6 gliomas (Benjamin and Keshet, 1997). Nitric oxide can induce VEGF expression and mediate the angiogenic effects of VEGF (Ambs *et al*, 1999). We therefore hypothesised that the reduced growth and vessel perfusion of AS7 tumours would be associated with decreased VEGF expression. However, our *in vitro* and *ex vivo* analyses of VEGF expression and production in tumour cells and explants revealed no significant differences between AS7 and C6 gliomas, coupled with no difference in vessel density as revealed by PECAM immunohistochemistry. Endothelial NOS has been shown to have a predominant role in VEGF-induced angiogenesis (Fukumura *et al*, 2001). This, coupled with the data herein, suggests that NO produced from iNOS activity is important in the maintenance of tumour blood vessel tone and function, and further highlights the apparent differing roles of the NOS isotypes, and their tissue/cell type of origin, on tumour angiogenesis *in vivo*.

In conclusion, we have shown (i) by using an antisense approach that NO derived from tumour cell expression of iNOS is a key modulator of tumour growth and angiogenesis, and (ii) the ability of susceptibility contrast MRI to non-invasively assess the effects of tumour-derived iNOS on vascular development and function in C6 gliomas *in vivo*. Inhibition of NOS following treatment with L-NNA has been shown to have antivascular activity in human tumours (Ng *et al*, 2007). Together, these data further support the concept of inhibiting iNOS as a therapeutic strategy for the treatment of cancer.
